# Case Report: A Pediatric Case of Familial Mediterranean Fever Concurrent With Autoimmune Hepatitis

**DOI:** 10.3389/fimmu.2022.917398

**Published:** 2022-06-24

**Authors:** Mariko Aoki, Kazushi Izawa, Takayuki Tanaka, Yoshitaka Honda, Takeshi Shiba, Yukako Maeda, Takayuki Miyamoto, Keisuke Okamoto, Masahiko Nishitani-Isa, Hiroshi Nihira, Kohsuke Imai, Junko Takita, Ryuta Nishikomori, Eitaro Hiejima, Takahiro Yasumi

**Affiliations:** ^1^ Department of Pediatrics, Faculty of Medicine, Kyoto University Graduate School of Medicine, Kyoto, Japan; ^2^ Laboratory of Lymphocyte Activation and Susceptibility to EBV Infection, INSERM UMR 1163, Imagine Institute, Paris, France; ^3^ Department of Pediatrics, Tokyo Medical and Dental University, Tokyo, Japan; ^4^ Department of Community Pediatrics, Perinatal and Maternal Medicine, Tokyo Medical and Dental University, Tokyo, Japan; ^5^ Department of Pediatrics and Child Health, Kurume University School of Medicine, Kurume, Japan

**Keywords:** familial Mediterranean fever (FMF), autoimmune hepatitis (AIH), liver injury, functional analysis, pediatric case report

## Abstract

Familial Mediterranean fever (FMF) is a hereditary, autoinflammatory disease that causes recurrent fever, arthritis, and serositis. The diagnosis of FMF is based on the presentation of typical clinical symptoms and the Mediterranean fever gene (*MEFV*) test. However, the challenge lies in diagnosing atypical cases. In this report, we have described a pediatric patient with complex FMF whose diagnosis required trio-whole exome sequencing (WES) and functional validation of a rare *MEFV* variant. A 3-year-old boy presented with recurrent episodes of elevated liver enzymes and arthralgia. He was diagnosed with autoimmune hepatitis (AIH), and his liver enzymes improved rapidly with steroid treatment. However, he exhibited recurrent arthralgia and severe abdominal attacks. Trio-WES identified compound heterozygous mutations in *MEFV* (V726A and I692del). *Ex vivo* functional assays of the patient’s monocytes and macrophages, which had been pre-treated with *Clostridium difficile* toxin A (TcdA) and colchicine, were comparable to those of typical FMF patients, thereby confirming the diagnosis of FMF. Although he was intolerant to colchicine because of liver toxicity, subsequent administration of canakinumab successfully ameliorated his abdominal attacks. However, it was ineffective against liver injury, which recurred after steroid tapering. Therefore, in this case, the pathogenesis of AIH was probably interleukin-1β (IL-1β)-independent. In fact, AIH might have been a concurrent disease with FMF, rather than being one of its complications. Nevertheless, further studies are necessary to determine whether FMF-induced inflammasome activation contributes to AIH development. Moreover, we must consider the possibility of mixed phenotypes in such atypical patients who present distinct pathologies simultaneously.

## Introduction

Familial Mediterranean fever (FMF) is a common, monogenic, autoinflammatory disease that is mainly characterized by periodic fever and serositis. Mutations in the Mediterranean fever gene (*MEFV)*, which encodes pyrin, are responsible for FMF. Mutant pyrin causes hyperactivation of inflammasomes and interleukin-1β (IL-1β) hypersecretion (1). Incidentally, FMF diagnosis might be difficult since it exhibits diverse clinical manifestations, and the majority of *MEFV* variants are classified as variants of unknown significance (2, 3).

Sometimes, FMF patients may develop non-amyloid liver diseases, such as nonalcoholic fatty liver disease and cryptogenic cirrhosis (4). Additionally, few studies reported the incidence of autoimmune hepatitis (AIH) in FMF patients (4–7). In this report, we describe the case of a patient with complex FMF, whose diagnosis was difficult because of concurrent AIH and which required functional analysis of a rare *MEFV* variant as well as trio-whole exome sequencing (WES).

## Case Description

**Figure 1:**
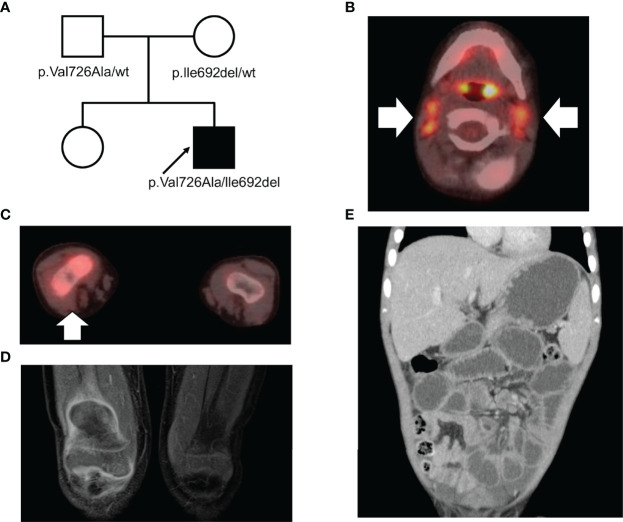
**(A)** A pedigree of the family. The arrow indicates the proband. **(B, C)** Positron emission tomography with computed tomography scanning in **(B)** the cervical lymph nodes and **(C)** the right knee joint. **(D)** Contrast-enhanced magnetic resonance imaging of the synovium of the right knee joint in T1 weighted image. **(E)** Computed tomography scan of the abdomen.

The patient was born to a Jewish (Eastern European ancestry) father and a Japanese mother (**Figure 1A**). There was no consanguineous marriage in the patient’s family. The patient’s mother developed neuromyelitis optica during her pregnancy with the patient, and his father subsequently developed minimal change nephrotic syndrome. He had been experiencing recurrent episodes of migratory arthritis that were sometimes associated with fever or elevated liver enzymes since he was 2 years old. The patient visited our hospital complaining of right knee arthralgia when he was 3 years old. Physical examination revealed bilateral cervical lymphadenopathy and swelling of the right knee joint. However, significant hepatosplenomegaly was not detected. Blood tests revealed elevated liver enzymes (aspartate aminotransferase 76 IU/L; alanine transaminase 87 IU/L) and indicated the presence of an inflammatory response (C-reactive protein 105 mg/L; erythrocyte sedimentation rate 63 mm/h). Furthermore, he exhibited hypergammaglobulinemia (IgG 1,987 mg/dL), elevated anti-smooth muscle antibody titer (1:80), and positive anti-liver kidney microsomal-1 antibody, all of which indicated an autoimmune disease. Tests for hepatitis B and C virus were negative. ^18^F-fluorodeoxyglucose positron emission tomography with computed tomography scanning demonstrated fluorodeoxyglucose accumulation in the cervical lymph nodes and right knee joint (**Figures 1B, C**). Moreover, magnetic resonance imaging revealed contrast enhancement along the synovium of the right knee joint (**Figure 1D**).

**Figure 2:**
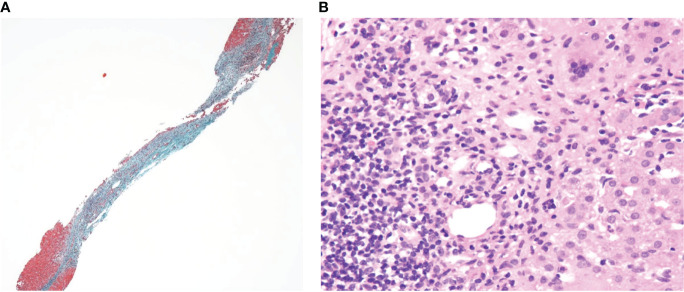
**(A)** Elastica-Masson staining (×40) and **(B)** Hematoxylin and eosin staining (×200) of the liver sections.

Incidentally, the aspartate aminotransferase/alanine transaminase levels elevated to 384/357 IU/L in 2 w, leading us to perform a liver biopsy. Liver pathology displayed fibrotic enlargement and lymphocytic infiltration in 50% of the portal areas with mild interface activity. Although plasmacytoid infiltration which is typical in AIH was not clear, the overall histopathological image was consistent with that of AIH (**Figures 2A, B**). According to the revised original scoring system of the International Autoimmune Hepatitis Group, our patient had a score of 14 prior to treatment, indicating a probable diagnosis of AIH (8). Liver enzymes spontaneously normalized in another 2 w, without treatment.

When the patient was aged 3 years and 4 months, he started experiencing severe abdominal attacks at an interval of a few days or weeks. Each attack lasted 1–3 d and was accompanied by fever as well as highly elevated inflammatory markers. During the attack, liver enzymes were elevated up to 20 times the normal upper limit. He was treated with methylprednisolone pulse therapy and azathioprine for the suspected AIH. Although liver enzymes were normalized rapidly owing to the prednisolone and azathioprine maintenance, recurrent abdominal attacks and arthritis persisted. Moreover, after reducing the prednisolone dose, liver enzymes were re-elevated. Magnetic resonance cholangiopancreatography, laparoscopic cholangiography, and upper and lower endoscopy were performed to assess the possibility of primary sclerosing cholangitis; however, no abnormalities were detected. Therefore, we concluded that the patient was experiencing a relapse of AIH, treated him with methylprednisolone pulse, and ensured a gradual reduction of the prednisolone dose this time.

**Figure 3:**
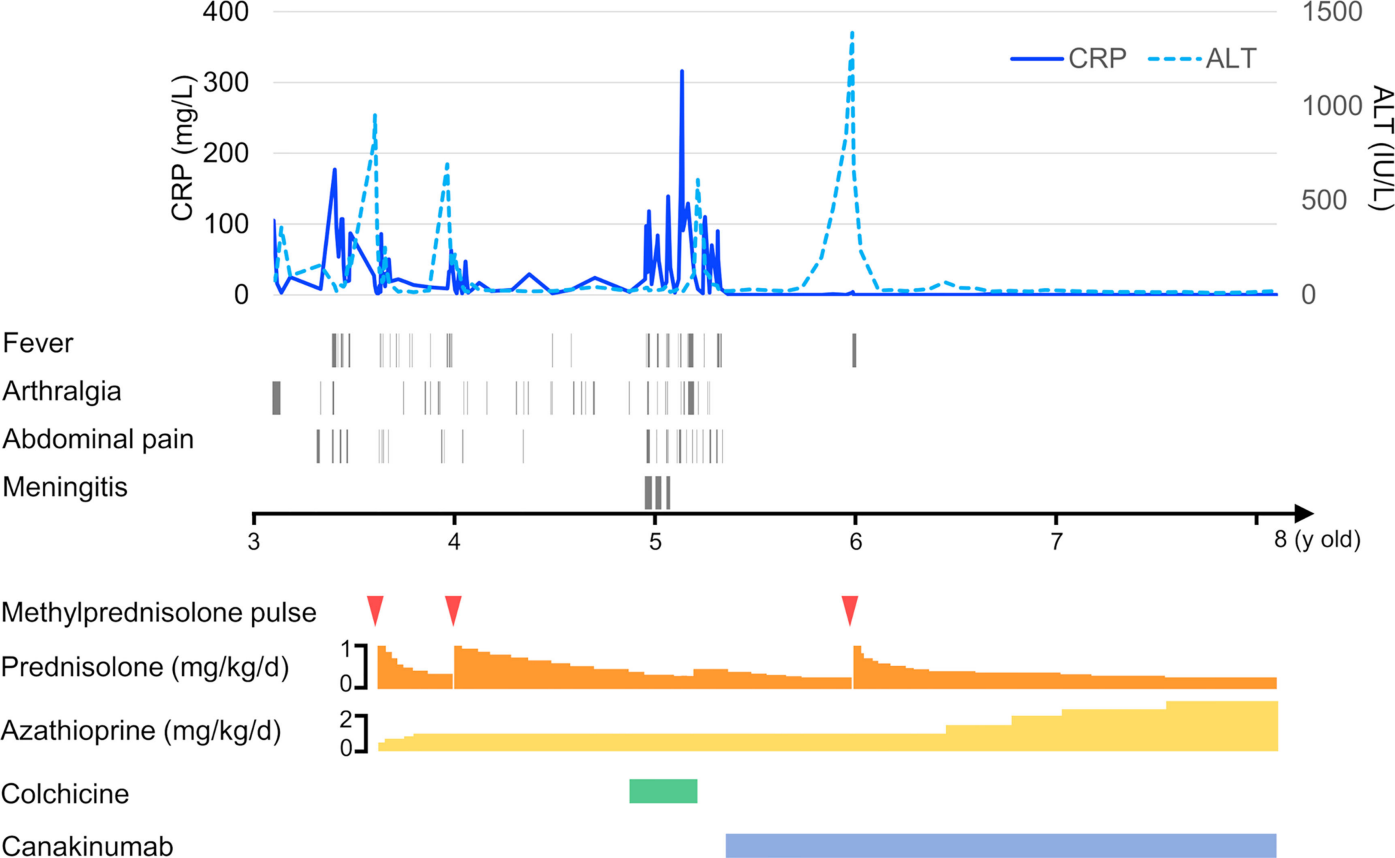
Timeline of the case exhibiting hematological testing, symptom presentation, and treatment.

Since fever and abdominal attacks are not typical AIH symptoms, we performed trio-WES and identified compound heterozygous mutations in *MEFV* (V726A and I692del). WES did not reveal any additional pathogenic variants of the known primary immunodeficiency genes. Subsequently, we performed *ex vivo* functional analysis of the *MEFV* variants using the patient’s peripheral blood (described below), which, in turn, confirmed the FMF diagnosis. We started low-dose colchicine therapy at 0.01 mg/kg/d, with special attention to liver toxicity when the patient was aged 4 years and 10 months. However, the patient developed a headache and vomiting. The cerebrospinal fluid white blood cell count was 641 cells/μL (mononuclear cells 21.7%, polymorphonuclear cells 78.3%). He was diagnosed with aseptic meningitis based on the absence of bacterial growth on the cerebrospinal fluid culture. Simultaneously, he experienced fever, abdominal pain, and arthralgia, suggesting that the meningitis was a manifestation of FMF. Unfortunately, the liver enzymes were elevated again at the age of 5 years and 2 months, indicating a possibility of colchicine-associated liver toxicity. Hence, the colchicine therapy was discontinued. Thereafter, the patient again experienced severe abdominal pain, following which a computed tomography scan revealed paralytic ileus and mural thickening of the small intestine (**Figure 1E**). Fortunately, his condition improved with conservative treatment. When he was aged 5 years 3 months, we introduced canakinumab (2 mg/kg every 4 w), which markedly ameliorated the frequency as well as the severity of the abdominal attacks. However, liver enzymes continued to elevate upon steroid tapering. Liver biopsy at the age of 5 years and 9 months revealed interface hepatitis with portal lymphocytic infiltrate, indicating AIH relapse. Incidentally, methylprednisolone pulse therapy promptly normalized liver enzymes, and he was free of FMF/AIH attacks for 2 y. (**Figure 3**). We plan to continue treatment with canakinumab for FMF, and steroids and azathioprine for AIH.

## Functional Analysis of Patient’s Peripheral Blood Mononuclear Cells (PBMCs)

**Figure 4:**
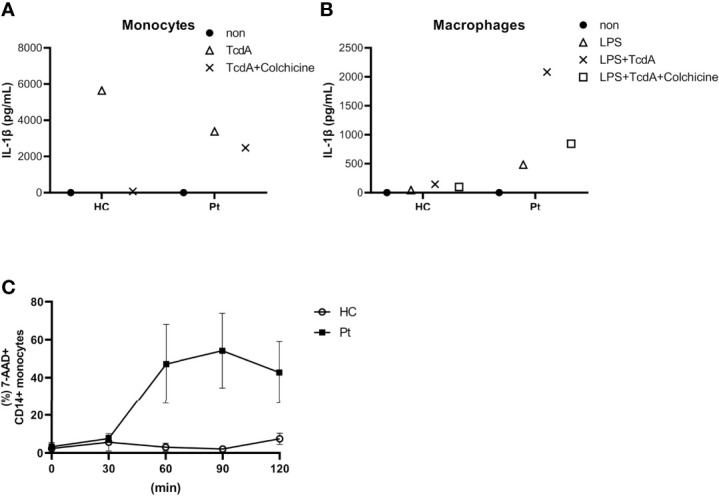
**(A)** Interleukin 1β (IL-1β) secretion from primary monocytes in response to *Clostridium difficile* toxin A (TcdA) with or without colchicine. **(B)** IL-1β secretion from LPS-primed monocyte-derived macrophages upon TcdA stimulation with or without colchicine. **(C)** Time course of UCN-01-induced monocyte cell death. Data were obtained from the patient (A/B; data from single experiment with technical duplicates for each condition, C; data from two independent experiments) and three healthy controls (HC).

Although V726A has been reported as a classical pathogenic mutation of *MEFV*, reports about the pathogenicity of the I692del variant are limited. To confirm the pathogenicity of the *MEFV* mutations identified by WES, we performed an *ex vivo* functional assay (9, 10). Interestingly, *Clostridium difficile* toxin A (TcdA)-induced IL-1β secretion from monocytes was inhibited by colchicine in healthy controls, but not in our patient (**Figure 4A**). Moreover, lipopolysaccharide (LPS) and TcdA-induced IL-1β secretion from peripheral blood-derived macrophages (PB-MP) was markedly enhanced in our patient, compared to that in healthy controls, which was partially suppressed by colchicine (**Figure 4B**). These response patterns were consistent with those of FMF patients, thereby supporting the diagnosis of FMF in our case. Moreover, UCN-01 treatment induced cell death in a time-dependent manner in our patient’s monocytes (**Figure 4C**), which was consistent with the general response shown by FMF patients; in fact, UCN-01 induces dephosphorylation of pyrin, inflammasome activation, and pyroptosis in monocytes of FMF patients (11).

## Discussion

In this report, we have described a pediatric case of FMF concurrent with AIH. Steroids and azathioprine improved the AIH symptoms of our patient but did not alleviate the febrile attacks, arthralgia, and abdominal pain. Trio-WES led to the diagnosis of FMF. Canakinumab therapy caused remission of FMF symptoms but could not prevent AIH relapse. Hence, in this case, the pathogenesis of AIH was probably IL-1β-independent, and the AIH was a concurrent disease with FMF, rather than being one of its complications. A possible explanation for the simultaneous occurrence of elevated liver enzymes and febrile attacks is that excessive inflammation due to pyrin inflammasome activation may exacerbate AIH. The prevalence of AIH is 2 per 2,000 FMF patients (5), which is higher than that in the general population of the United States, Denmark, and Israel (31.2, 23.9, and 11.0 per 100,000 individuals, respectively) (12–14). Although many studies have described the association between FMF and liver injury, only four cases of FMF-associated AIH have been reported to date (4). However, the details of these cases have not been described, and the direct involvement of FMF in AIH development has not been elucidated (5–7). Hence, it is not necessarily true that the incidence of AIH is high solely due to excessive inflammation caused by activation of pyrin inflammasomes. A recent study showed that IL-1β production increased depending on the activation of NLRP3 inflammasome in a concanavalin A-induced AIH murine model (15). However, to the best of our knowledge, the association of *MEFV*-encoded pyrin inflammasome with AIH has not yet been reported. Therefore, it is difficult to determine the pathogenic mechanism of concurrent AIH with FMF based on only this case. Further studies are needed to determine whether the inflammasome activation in FMF contributes to AIH development.

Usually, FMF presents clinical symptoms namely recurrent fever and serositis, and its occurrence is confirmed by genetic analysis. However, due to the variants of unknown significance in *MEFV*, an accurate diagnosis of FMF might be difficult. In this case, trio-WES identified unexpected, compound heterozygous mutations in *MEFV*. Unfortunately, only a few reports describe *MEFV* ^I692del^, and its function has not been completely analyzed (16, 17). Recently, the pathogenicity of 32 *MEFV* variants including *MEFV* ^I692del^ was evaluated by enhancing cell death in *MEFV* variant-transfected THP-1 cells, and comparing them to *MEFV* ^WT^-transfected cells. The response of *MEFV* ^I692del^ was similar to that observed in cells transfected with *MEFV* ^M694V^, the classical *MEFV* mutation (3). This result is consistent with our analysis. Therefore, functional analysis of *MEFV* variants along with an assessment of the patient’s peripheral blood can help to diagnose FMF.

It is important to differentiate potentially life-threatening small-bowel obstructions from FMF-associated abdominal attacks since the latter does not require surgical intervention. However, recurrent peritonitis in FMF might result in peritoneal adhesions, which, in turn, cause small bowel obstruction (18). A previous study showed that 11 out of 355 pediatric FMF cases exhibited adhesive small bowel obstruction within a mean of 3.7 y from diagnosis (19). Incidentally, if the patient exhibits only paralytic ileus, then conservative treatment may be effective, as observed in this case. However, if the bowel is obstructed, then emergency surgery might be necessary to avoid the risk of intestinal necrosis. Although the initial abdominal pain makes it difficult to differentiate between these two conditions, a prolonged and/or severe abdominal pain that is different from the “usual” pain pattern is a critical alert for bowel obstruction (18). If the patient experiences such an “unusual” pain, a computed tomography scan for proper diagnosis needs to be considered.

In conclusion, we have described a pediatric FMF case with an atypical presentation. Owing to the initial presentation as AIH, we did not suspect the incidence of FMF. Nevertheless, we were able to confirm FMF diagnosis using trio-WES and *ex vivo* functional analysis. In such cases, to provide appropriate treatment for each disease, we must consider the possibility of a mixed phenotype in atypical patients presenting distinct pathologies simultaneously.

## Materials and Methods

Peripheral blood mononuclear cells were isolated using Lymphoprep (Alere Technologies, Waltham, MA, USA). Among them, CD14+ monocytes were sorted magnetically using AutoMACS Pro Separator (Miltenyi Biotec, Bergisch Gladbach, Germany), according to the manufacturer’s instructions. PB-MPs were generated, based on a previously described protocol (10). CD14+ monocytes were cultured in RPMI-1640 medium (Sigma-Aldrich, St. Louis, MO, USA) supplemented with 10% fetal bovine serum (Gibco, Carlsbad, CA, USA) and 50 ng/mL macrophage-colony stimulating factor (R & D Systems, Minneapolis, MN, USA) for 7 d to obtain PB-MPs.

Monocytes were seeded in flat-bottom 96-well plates (Falcon, Corning, NY, USA) at 5 × 10^4^ cells/well in RPMI-1640 medium supplemented with 10% fetal bovine serum. Cells were pretreated with colchicine (100 ng/mL; Sigma-Aldrich) for 30 min, as indicated, followed by the addition of 1 μg/mL TcdA (Enzo Life Sciences, Farmingdale, NY, USA). Culture supernatants were collected after 4 h of incubation. Similarly, macrophages were seeded in 96-well plates. Cells were pretreated with colchicine for 30 min, as indicated. After 2 h of priming with 1 μg/mL LPS (*In vivo*Gen, San Diego, CA, USA), the cells were treated with 1 μg/mL TcdA for 4 h. Culture supernatants were collected. The IL-1β secretion was analyzed in both the supernatants using the Bio-Plex MAGPIX system (Bio-Rad Laboratories Inc. Hercules, CA, USA).

To analyze UCN-01-induced monocyte cell death, the peripheral blood mononuclear cells were resuspended in RPMI-1640 medium with 10% fetal bovine serum at 2.5 × 10^6^ cells/mL and stimulated with UCN-01 (10 μM, Sigma-Aldrich), as indicated. Monocyte death was quantified as the percentage of 7-aminoactinomycin D (BD; Becton and Dickinson Bioscience, Franklin Lakes, NJ, USA)-positive, annexin V (BD)-positive cells among CD14 (Biolegend, San Diego, CA, USA)-positive monocytes using fluorescence-activated single-cell sorting verse (BD).

## Data Availability Statement

The raw data supporting the conclusions of this article will be made available by the authors, without undue reservation.

## Ethics Statement

The studies involving human participants were reviewed and approved by Kyoto University Graduate School and Faculty of Medicine, Ethics Committee. Written informed consent to participate in this study was provided by the participants’ legal guardian/next of kin. Written informed consent was obtained from the individual(s), and minor(s)’ legal guardian/next of kin, for the publication of any potentially identifiable images or data included in this article.

## Author Contributions

MA wrote the case report and prepared the figures. KIz and EH were the attending physicians of this patient and the director of the whole writing process. TT, YH, TS, YM, TM, KO, MN, HN, KIm, JT, RN, and TY performed the diagnostic evaluation of the patient. TT, YH, and TS performed the laboratory evaluation for the diagnosis. All authors contributed to the article and approved the manuscript.

## Funding

This research was supported by a Health Labor Sciences Research Grant for Research on Intractable Diseases from the Ministry of Health, Labor and Welfare (MHLW) of Japan (H29-Nanchi-Ippan-020 and JPMH20317089 to KIz, RN, EH, and TY).

## Conflict of Interest

The authors declare that the research was conducted in the absence of any commercial or financial relationships that could be construed as a potential conflict of interest.

## Publisher’s Note

All claims expressed in this article are solely those of the authors and do not necessarily represent those of their affiliated organizations, or those of the publisher, the editors and the reviewers. Any product that may be evaluated in this article, or claim that may be made by its manufacturer, is not guaranteed or endorsed by the publisher.
